# Cancer Stem Cell Radioresistance and Enrichment: Where Frontline Radiation Therapy May Fail in Lung and Esophageal Cancers

**DOI:** 10.3390/cancers3011232

**Published:** 2011-03-10

**Authors:** Giang Huong Nguyen, Mandi M. Murph, Joe Y. Chang

**Affiliations:** 1 Balliol College, University of Oxford, Oxford, UK; E-Mail: giang.nguyen@balliol.ox.ac.uk; 2 Department of Pharmaceutical and Biomedical Sciences, College of Pharmacy, University of Georgia, Athens, Georgia 30602, USA; E-Mail: mmurph@rx.uga.edu; 3 Department of Radiation Oncology, The University of Texas M.D. Anderson Cancer Center, Houston, Texas 77030, USA

**Keywords:** cancer stem cells, lung cancer, esophageal cancer, radiation therapy

## Abstract

Many studies have highlighted the role cancer stem cells (CSC) play in the development and progression of various types of cancer including lung and esophageal cancer. More recently, it has been proposed that the presence of CSCs affects treatment efficacy and patient prognosis. In reviewing this new area of cancer biology, we will give an overview of the current literature regarding lung and esophageal CSCs and radioresistance of CSC, and discuss the potential therapeutic applications of these findings.

## 1. Introduction

Cancer has become the leading cause of human death worldwide [1]. Despite the recent advances made in cancer treatment and diagnosis, cancer mortality for advanced solid malignancies remains unchanged, in part due to the development of resistance by the tumor to radiation and chemotherapy. One possible cause for therapeutic failure is that residual tumor cells are reminiscent of stem cells, which ultimately give rise to secondary tumors or distant metastasis. From a therapeutic perspective, the state of knowledge regarding cancer stem cells will help to advance the development of treatments directed against this population of cancer cells.

Stem cells are present in many different somatic tissues and have three distinctive properties: self-renewal, the ability to develop into multiple lineages, and the potential to proliferate extensively [2]. Recent studies have defined a subset of tumor cells found in breast [3], brain [4] hematopoietic [5], colon [6], pancreatic [7], stomach [8], liver [9], skin [10], ovarian [11], bladder [12], head and neck [13], and most recently lung [14], that also possess these three features which are parallel to stem cells. Although lung and esophageal cancers are among the most common lethal forms of cancer in both men and women in the world, with the overall five-year survival rate at less than 20% [1], comparatively little is known about the biology of these two types of cancer stem cells compared to other solid tumor stem cells, particularly esophageal cancer. Therefore, there is an increasing need to understand the biology of lung and esophageal cancer stem cells so that new therapeutic developments could tackle this cell population.

Both lung and esophageal cancers are considered complex tumors in terms of their origin and regionally distinct types of neoplasia. Lung cancers are comprised of two major histological types: small-cell lung cancer (SCLC) and non-small-cell lung cancer (NSCLC), which can be further divided into squamous cell carcinoma (lung SCC), adenocarcinoma (lung ADC) and large cell carcinoma. Among these different types of lung cancer, lung ADC is the most common form of lung cancer in both smokers and nonsmokers. Lung SCC has a 25% prevalence rate and is commonly associated with tobacco smoking. Compared with other types of lung cancer, large cell carcinoma belongs to a class of poorly differentiated and less aggressive tumors. SCLC accounts for more than 20% of lung tumors in the population, and despite its initial response to therapy, it has a particular poor prognosis due to its highly metastatic, potential drug-resistance, and rapidly fatal behavior [15].

Like lung cancer, esophageal cancer is composed of two main histological subtypes: squamous cell carcinoma (SCC), mainly caused by cigarette smoking, and adenocarcinoma (ADC), mainly caused by gastric reflex and obesity. The incidence of ADC has increased remarkably over the past two decades and has supplanted SCC as the dominant phenotype in Western countries [16]. ADC frequently arises from Barrett's esophagus (BE), a chronic inflammatory condition characterized by a change in the normal esophagus epithelium into intestinal metaplasia of gastroesophageal reflux [17] and genomic instability [18]. The histological and regional diversity found in lung and esophageal cancer may partly be due to the presence of diverse pools of stem cells with different biological properties, underlining a pressing need to characterize these different stem cells for direct implications on diagnostic and therapeutic outcome.

## 2. Cancer Stem Cell Markers

A variety of approaches has been employed to isolate CSCs, often based on markers characteristic of normal stem cells such as CD44 [19], CD133 [20], CD15 [21], CXCR4 [22], *etc*. Although the list of CSC markers is not yet completed, they provide a means to isolate and substantially enrich CSCs for cancer research. In this review, we chose to focus our attention to CSC markers that are relevant to lung and esophageal tumors (Table 1).

One of the most common markers found on many of the CSCs identified to date is CD133. Although its exact function remains poorly understood, the expression of CD133 has been detected in many different types of cancer cells, including lung cancer [23]. CD133 is a highly conserved antigen that is a five-transmembrane domain cell surface glycoprotein [24]. It is a ubiquitous marker of CSCs since its expression is presented on a wide spectrum of tumors including brain [4], colorectal [6], pancreatic [25], breast [26], prostate [27], ovarian [28], liver [29], hematopoietic [30] and lung cancer [23]. CD133 was first identified in hematopoeitic stem and progenitor cells [31].

Cells isolated from primary lung tumors that are CD133^+^ show increased tumorigenicity and expression of stemness, adhesion, and drug efflux genes compared with the corresponding CD133^−^ tumor cells [32]. It is striking that CD133^+^ lung cancer cells survive cisplastin administration in either *in vitro* drug exposure on A549 cells or *in vivo* primary tumor-derived mouse xenografts [32]. This suggests that the biological differences between cancer cells and CSCs confers the CSCs with chemoresistance, which has enormous, paradigm-shifting implications regarding patient treatment since cisplatin is a first-line agent in both lung and esophageal cancers [33]. Evidence to suggest that CSCs have an impact on survival, possibly through platinum-resistance mechanisms, includes data that the five-year survival rate of CD133^+^ patients was significantly lower than that of CD133^−^patients; furthermore, the expression of CD133 is reportedly an independent prognostic factor in some studies, thus providing the first evidence of the importance of CSC markers as potential diagnostic and prognostic indicators in lung cancer patients [34].

In contrast, whether CD133 is a prognostic factor remains unclear since CD133 is not detected among all lung cancer samples [35]. Furthermore, Salnikov *et al*. reported that although CD133 expression is abundant in NSCLC, it is not a prognostic indicator for NSCLC patients [36]. Additional evidence has also indicated that some CD133-negative lung CSCs also possess the ability to self-renew and generate robust xenograft outgrowth [37], again suggesting that not all lung CSCs have CD133 as a marker. Unlike lung CSCs, CD133 expression was not present in cancer stem cells isolated from NOD-SCID mice that received transplantation of esophageal adenocarcinoma cells [38].

Another common marker for the identification of CSCs is CD44, which was originally described as a leukocyte-homing receptor, is involved in cell-cell interaction and is a downstream target of the Wnt/β-catenin pathway [39]. It comprises a family of glycoproteins, all encoded by a single gene, but varying in size due to alternative splicing [40]. Its expression was first identified in breast CSCs as these cells seem to express CD44 variant isoforms, rather than a CD133^+^ population [3]. CD44 expression has then been identified in many types of CSCs including SCLC and NSCLC and its expression is correlated with survival [41]. CD44 expression has been found in esophageal SCC and its expression is also known to correlate with poor prognosis [42-43].

An alternative method to isolate stem cells is to rely on the ability of ABC transporters expressed in stem cell populations, to efflux the fluorescent Hoechst 33342 dye [44]. This population of cells with enhanced efflux capabilities is known as Side Population cells (SP cells), which have been described in many tumor types as being rich in stem-like properties, including those of lung and esophageal cancer [45]. In both human lung and esophageal cancer, as few as 1000 isolated SP cells could produce robust xenografts in mice whereas a higher number of non-SP cells are required to generate tumors [46-47], sometimes as high as 5 × 10^4^ or 5 × 10^5^. Interestingly, in both lung and esophageal cancer, the SP population cells are less sensitive to chemotherapy than non-SP cells [46-47].

SP cells are also found to self-renew and express elevated levels of other so-called stemness genes such as hTERT (telomerase) [46], Oct-4 [48], SOX2 [49], BMI [47] and ZFX [47], as well as genes that are components of the Wnt and β-catenin pathways [50]. Oct-4 is known to play an important role in cell viability, functions as a stem cell survival factor, and induces an induction of pluripotency in somatic cells [51]. In addition, Oct-4 plays a crucial role in maintaining self-renewal, CSC-like and chemo- and radio-resistant properties of CD133^+^ NSCLC cells [52]. In squamous cell carcinoma of the esophagus, Oct-4 expression is highly elevated in the SP population compared to non-SP cells [47].

Another marker, aldehyde dehydrogenase (ALDH) is a detoxifying enzyme, known for its role in the oxidation of intracellular aldehydes, which play a role in stem cell differentiation through metabolism of retinal to retinoic acid [53]. ALDH is highly expressed in the tumorigenic cell population of various cancers including breast [54], brain [55], colon [56], head and neck [57]. The first evidence indicating ALDH is a relevant lung CSC marker is elevated ALDH protein expression discovered in putative lung stem cell niches during malignant transformation [58]. Further evidence to support ALDH as a functional marker came from Jiang's group who reported that NSCLC cancer cells with strong ALDH1 activity showed CSC features and were positive for CD133+ [59-60]. In addition, expression of ALDH1 is positively correlated with stage and grade of lung tumors and related to poor prognosis in patients with early-stage lung cancer [58]. In esophageal cancer, patients with deficient ALDH showed high risk for developing cancer, although there is no report to date about the existence of ALDH1 in esophageal CSC [61].

## 3. Signal Transduction Pathways in Cancer Stem Cells

WNT, Notch, Hedgehog and BMP signaling pathways constitute the stem cell signaling network which regulates the balance of self-renewal, proliferation and differentiation among stem and progenitor cell populations [62]. β-catenin is an essential component of both intracellular junctions and the canonical Wnt signaling pathway, which has been implicated in stem cell survival [63]. Earlier observations showed that overexpression of β-catenin could promote CSC survival and tumorigenesis both *in vitro* and *in vivo* [64], suggesting an important role for the Wnt/β-catenin pathway in the maintenance and regulation of CSC self-renewal. Recently, a study by Stripp *et al*. suggested that activated Wnt/β-catenin signaling in the developing lung coincided with an expansion of bronchioalveolar stem cells and attenuated their differentiation [65]. Additionally, evidence suggested that activated Wnt signaling in lung tumors promoted tumorigenesis [66,67]. However, the role of Wnt/β-catenin signaling in lung CSCs might be different for NSCLC than for SCLC. For example, aberrant Wnt pathway has been shown to play a role in NSCLC such as Wnt2 is overexpressed in NSCLC and inhibition of Wnt2-mediated signaling leads to apoptosis in NSCLC cell lines [68].

There are secreted Wnt antagonists that interact directly and indirectly to modulate Wnt signaling, therefore influencing tumorigeneis [69]. A secreted Wnt antagonist, Wnt inhibitory factor (WIF1), has been shown to inhibit NSCLC growth both *in vitro* and *in vivo* [70]. Interestingly, the Wnt inhibitor Dickkopf-1 (Dkk-1) is expressed in distal pulmonary epithelium and studies have shown that knocking out Dkk-1 inhibits branching morphogenesis [71]. In esophageal ADC cancer, Dkk-1 expression increases significantly starting with normal esophagus epithelium and low grade dysplasia to high grade dysplasia and esophageal adenocarcinoma [72]. Additionally, Dkk-1 expression is highly elevated in tumor samples obtained from esophageal SCC patients and its expression is a predictor of poor survival [73]. Reduced β-catenin expression level also correlates with poor prognosis in esophageal SCC patients [74].

Notch signaling plays fundamental roles in defining cell fate, self-renew, and is frequently deregulated in human malignancies [75]. In lung and esophageal CSCs it is not yet clear if Notch signaling is required for self-renewal, although several reports suggested that components of the Notch signaling network are expressed in putative lung CSC populations [32,76]. Knockout mouse studies demonstrate a requirement for Notch signaling in lung development [77,78]. Elevated Notch ligand receptor, and its transcriptional factor, *HES1* levels have been demonstrated in NSCLC lines [79,80] and Notch signaling also seems to be among key downstream effectors of oncogenic RAS [81], which is a common aberration observed in lung cancer. In addition, activation of Notch-1 in A549 adenocarcinoma cells inhibits the *in vitro* clonogenicity and *in vivo* tumorigenicity growth in mice, highlighting the complexity of the Notch pathway and its varying roles in different lung tumor subtypes [82]. Notch receptor expression is rarely seen in SCLC, likely because overexpression of activated Notch receptors inhibits SCLC growth [83]. Unlike what is seen in lung cancer, Notch signaling is inactivated in esophageal SCC, acting in an anti-oncogenic manner [84].

The Hedgehog pathway is also indispensable for normal mammalian embryo and organogenesis [85]. Although it is not yet clear if both lung and esophageal CSCs require Hedgehog signaling for self-renewal, several studies have suggested that specific inhibitors targeting the Hedgehog pathway could hamper tumor growth, some of which are currently in clinical trails for lung SCLC [86,87]. Activation of this signaling pathway occurs through the binding of the Sonic Hedgehog (Shh), Indian Hedgehog (Ihh) and Desert Hedgehog (Dhh) morphogens to their receptor Patched, subsequently inhibiting the repression of Smoothened [88]. Shh-null mice exhibit hypoblastic lung buds without airway branching [89]. On the other hand, transgenic overexpression of Shh leads to the absence of functional alveoli and hyperproliferation of epithelial and mesenchymal pulmonary cells [90]. The growth of SCLC cell lines was inhibited by KAAD-cyclopamine, a small molecule inhibitor of Smoothened and therefore the Hedgehog pathway. These results, however, were not observed in NSCLC cell lines [91]. In esophageal SCC tumors, elevated expression of Hedgehog target genes was observed and treatment of esophageal cancer cells with cyclopamine reduced cell growth and induced apoptosis [92].

These are by no means a complete summary of the most current knowledge about CSC markers signal transduction pathways in lung and esophageal cancer but they support the notion that CSCs are present in lung and esophageal cancer. Many new studies are published regularly that further expand the knowledge base of this area. Even so, additional studies are still needed to understand the pivotal role of these CSC markers in order to further elucidate the mechanisms regulating the origin and maintenance of CSCs and to delineate differences between normal stem cells and CSCs that could be exploited in the treatment and diagnosis of lung and esophageal cancer.

## 4. Radiation Resistance of Cancer Stem Cells

Radiation therapy plays an important role in treating esophageal and lung cancers as a part of combined modalities treatment with chemotherapy and/or surgery. These treatment approaches have improved local control and survival rates with acceptable toxicity. However, recurrence is essentially seen in most patients and some patients develop radioresistance that might limit any further treatment, demonstrating a strong need for new treatment therapies and directions in the management of these two cancers.

**Figure 1.**
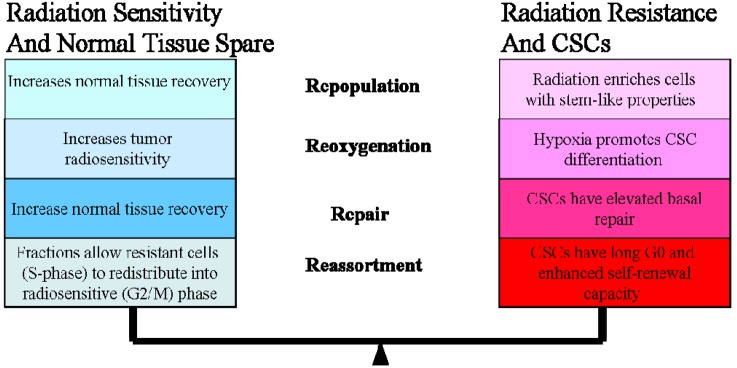
The potential complications of cancer stem cells for fractionated therapy.

In the clinic, radiotherapy is always applied in multiple fractions, a concept that is based on the knowledge of classical “4 R's of Radiobiology” among fractionations: (1) The repair of sub-lethal damage and (2) repopulation of non-cancerous cells; (3) reoxygenation and (4) reassortment of tumor cells in radiosensitive phases [93]. In this review, we offered a systematic review of CSC research pertaining to the 4 Rs. It is difficult to summarize all the current CSC literature addressed the 4 Rs, and in many cases there are conflicting data, but we have attempted to focus on the delicate balance between CSC radiosensitivity *vs*. radioresistance (Figure 1).

Ionizing radiation leads to cell death by production of unrepairable DNA double-strand breaks (DSBs). Most radiation-induced DNA damage, however, is sublethal as cells are able to repair damage at lower doses. At higher dose accumulation, these sublethal lesions contribute to lethality. (The 4R's of radiation biology: Reassortment of cells within the cell-cycle, Repair of sub-lethal damage, Reoxygenation, and Repopulation.)

The ability of cells to repair the sublethal damage is different between normal and tumor cells, a concept that forms the basic foundation for using multiple fractions in radiotherapy treatment planning [94]. However, this conventional understanding of radiation-induced damage needs to be reevaluated with a specific emphasis on CSCs (Figure 1).

A hallmark of DNA DSB recognition and repair is histone H2A phosphorylation, which is thought to mark sites of DNA damage [95]. Radiation induced few or significantly fewer γ-H2AX foci in human breast CSCs, and in mouse breast CSCs these resolved faster than in non-CSCs [96-97]. Also, in glioma, radiation induced DNA damage to a similar degree in CD133^+^ and CD133- cells, but CD133^+^ cells repaired DNA damage more efficiently and rapidly than CD133- cells [98]. Interestingly, CD133^+^ cells showed basal activation of Rad17, a component of the DNA damage checkpoint, suggesting that cancer stem cells are primed to respond to genotoxic stresses. Inhibition of this response radiosensitized CD133^+^ glioma cells [98]. Although these studies focus on glioblastoma and breast CSCs, we expect that these findings may be more broadly applicable to lung and esophageal cancer types. It would be interesting to see if the checkpoint activation in CD133^+^ cells is biologically important in lung and esophageal CSCs.

Accelerated repopulation of tumor cells during or after radiotherapy treatments are well documented as causes of treatment failure. Recent studies showed that irradiation enriches the fraction of cells expressing CSC markers [25,98]. For example, radioresistant esophageal carcinoma cells (Eca109R50Gy) obtained through fractionated irradiation (FIR) showed enhanced CSC properties and tumorigenic ability, approximately 40-times higher than that of radiosensitive cells in an *in vivo* xenograft tumorigenicity assay [99]. Interestingly, β-catenin expression spiked immediately with increasing irradiation doses and when the expression of β-catenin was suppressed by Wnt antagonist Dkk1, Eca109R50Gy cells displayed an enhancement of radiosensitivity [99]. In another study, Zhang *et al*. reported that several of the putative stem cell markers such as β-catenin, Oct3/4 and β-integrin were significantly increased in two radioresistant esophageal cell lines [100]. They also noticed that several so-called stemness genes were up-regulated while apoptotic genes were down-regulated in these cells. In addition, sorted CSCs from different types of tumors survived chemo-radiation treatments in culture much better than unsorted or negative cells [98]. This observation is supported by evidence that CSCs have high free radical scavenger levels [101], low proteasome activity [102], activated DNA checkpoints [10], slow cell-cycle progression [103-104], and efficient DNA repair machinery [98]. These factors are potential resistance mechanisms used by CSCs; highlighting the need for developing a revised radiation treatment protocol that could eliminate a small but resilient subpopulation of CSCs.

Radiation is also given on the assumption that tumors when oxygenated form highly reactive oxygen species (hROS), which rapidly interact with various biomolecules in cells, thereby causing DNA damage. There is ample evidence in the literature showing that hypoxic cells are more resistant to radiation than non-hypoxic cells [105], which would be an issue with many solid tumors. Highlighting the severity of this clinical problem is data demonstrating that human tumors containing regions of acute and chronic hypoxia are associated with poor prognosis because of local recurrence or systemic disease [106-108]. Reoxygenation between dose fractions is generally believed to improve the efficacy of radiation treatment by increasing tumor radiosensitivity. However, emerging evidence has indicated that hypoxic environment could actually trigger CSC differentiation; therefore leading to radiation resistance [109,110]. Given that CSCs have been shown to have low hROS metabolic profile, slow cycling, and express completely different survival pathways, it would be of great interest to know more about the response of CSCs to radiation under varying hypoxic conditions. Moving forward it would also have important clinical implications and therapeutic translation.

An early observation that shows CSCs are protected by hypoxia suggests this occurs through the induction of hypoxia-inducible factors (HIFs). Activation of HIF2α induces the expression of Oct-4, which is a central player in CSC self-renewal [111]. In addition, HIF2α is known to negatively interact with Notch1, therefore contributing to the maintenance of CSC undifferentiated state [112]. Another interesting point is that CSCs exist in two different states, one hypoxic for quiescent CSCs and one adjacent to endothelial cells for more activated CSCs. Transition between both states could be bidirectional, as is shown in hematopoietic stem cells [109]. These two states of CSC existence in relation to hypoxia might help to explain the variable results of clinical trials aimed at improving tumor oxygenation [113], as frequent hypoxia-reoxygenation caused by FIR could allow CSCs to differentiate and populate.

Each individual cell's position in the cell cycle influences its radiosensitivity, with cells in mitosis being most sensitive to DNA damage and cells in late S-phase most resistant [114]. Because of cell cycle progression between radiation fractions, dose fractionation allows redistribution of radioresistant S-phase tumor cells into a more sensitive phase of the cell cycle which allows maximal killing of the tumor cells while limiting toxicity to the surrounding healthy cells [115]. However, emerging evidence suggests that normal tissue stem cells and CSCs exist in the G_0_-phase of the cell cycle; therefore progressing slowly through the cell cycle [103,104,116] or maintaining a quiescent state for some period of time. Recent data on neural stem cells even suggest the possibility that expression of CD133 depends on the cell cycle and CD133 expression is specifically downregulated in the G_0_/G_1_ phase [98]. If this is true, it could explain why some lung cancer cells show little expression of CD133 but have the potential to self-renew [35]. Both lung cancer cell lines and human lung cancer samples contained a significant number of SP cells. These CSCs expressed a lower level of MCM7, a member of the minichromosome maintenance protein complex which is an essential component of the replication helicase complex, and is therefore a marker for S phase [46,117]. The human radioresistant esophageal cancer cell line, Eca109R50Gy had increased G_0_/G_1_ phase proportion, decreased G_2_/M phase proportion, and lower apoptosis rate compared with its parental cells, indicating they are relatively quiescent [99].

Another intriguing point, although it has not yet been seen in lung/esophageal samples, is that fractionated irradiation of breast CSCs induce elevated developmental signaling pathways like Notch/Jagged expression to a greater extent than single doses [96]. This data suggests that fractionated irradiation may specifically induce CSC differentiation through specific activation of different developmental pathways. If, in fact, tumors show a high percentage of slowly cycling CSCs and fractionated irradiation allows CSC differentiation, we may consider adjusting our current radiotherapy fractionation schedules to capture CSCs during their most vulnerable point in the cell cycle to induce DNA damage.

In summary, CSCs are more resistant to radiation therapy than non-stem cells. The cells that survive radiation may be enriched for more stem cells and actually exacerbate recurrence and cause refractory disease. For these important reasons, innovative radiotherapy approaches targeting CSCs might be the key to completely eradicating lung and esophageal tumors. Thus, the current understanding of radiotherapy and its treatment protocol need to be reexamined.

## 4. Implication for Cancer Research and Development

Despite enormous research efforts, the prognosis for both lung and esophageal cancer remains among the poorest. Surgery and radiation therapy are currently the only treatment options leading to a cancer cure for patients suffering from localized cancers. However, most current strategies may not target CSCs specifically but rather more differentiated tumor cells. Existing paradigms for cancer treatment need to be re-evaluated for relevance based upon recent findings of CSCs. One aspect that would greatly improve the current stage for treatment of lung and esophageal cancer is by further characterizing CSC markers seen in these two cancers. Although there is ample evidence of CSC markers in other solid tumors, there is little evidence on the specific CSC markers for respective subtypes of lung and esophageal cancers and the data reported sometimes conflict and can not be standardized to allow comparison across laboratories. Developing CSC markers for use in the clinic would facilitate the visualization of specific CSCs and the capture of related images used for treatment planning, which would be critical to strategically target these CSCs and reduce disease recurrence. Currently radiation doses are often prescribed on the basis of several clinical parameters including tumor volume, histology and location under the consideration of tolerance limits of the surrounding anatomical structures. Therefore, knowledge about the spatial distribution of CSCs in relation to non-stem cells within the tumor bed may become important during treatment planning. Therefore, this is an important area of research that needs to be actively examined before meaningful adjustments to current treatments can be taken.

The currently used imaging techniques for treatment planning are inadequate in discriminating between areas with active CSCs and those with non-stem cells. For instance, positron emission tomography (PET) or PET/CT with 2-deoxy-2-[18F]fluoro-D-glucose (^18^FDG-PET) is widely utilized for diagnosis, staging and assessment of therapeutic response during and after radiation therapy for lung/esophageal cancers [118,119]. This technology works on the basic assumption that rapidly proliferating cancer cells, which form the bulk of the tumor, rely mainly on glycolysis, a biochemical process that requires drastically increased glucose uptake; therefore, these cells will be seen on ^18^FDG-PET imaging of lung tumors or image-guided radiation therapy. Given what is known about CSCs, it is widely assumed that CSCs, in contrast, are mostly quiescent and metabolize glucose by oxidative phosphorylation rather than by glycolysis [120]. Therefore, areas with high numbers of quiescent CSCs with low glucose uptake may not be picked up by ^18^FDG-PET and therefore might not be targeted specifically during treatment planning. Thus, to improve the clinical relevance of PET/CT in tumor imaging, it is important to compliment these imaging techniques with an evaluation of CSCs. An area with enriched CSCs may in fact need a higher radiation dose to eliminate CSCs and is crucial to reduce disease progression and distant metastasis. This concept is supported by a recent clinical trial enrolling early stage lung cancer patients. These patients showed a promising clinical outcome with improved survival and local control (>95% compared with 50% using conventional radiotherapy to 60-70 Gy with 2 Gy/fraction) when hypofractionated stereotactic ablative radiotherapy, *i.e.*, higher dose per fraction (>10 GY) to total dose of 48 to 54 Gy, was delivered [121].

Recently, intensity-modulated radiation therapy (IMRT) was developed to create more conformal radiation dose to target while minimizing dose to surrounding critical structures and allowing selectively painting the dose to subregions within the target and further customizing delivered dose distribution [121]. Given the inhomogeneity of the tumor and the presence of CSCs, it would be extremely helpful to use CSC markers as biomarkers to guide IMRT treatment when designing a comprehensive treatment plan to improve biological conformality based on CSCs. Combining these two approaches could further improve the efficacy of future radiotherapy.

**Figure 2.**
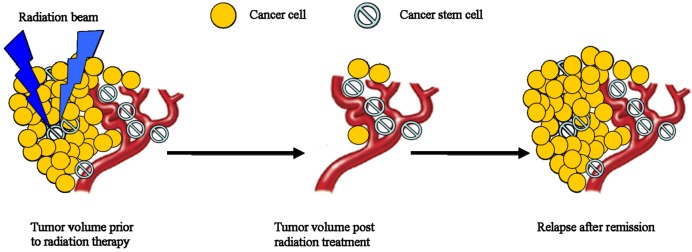
Conventional radiation treatment may target mainly the bulk of the tumor rather than the cancer stem cells (CSCs) in the perivascular niche. The CSCs that remain after radiation therapy can differentiate and subsequently form a new tumor.

**Figure 3.**
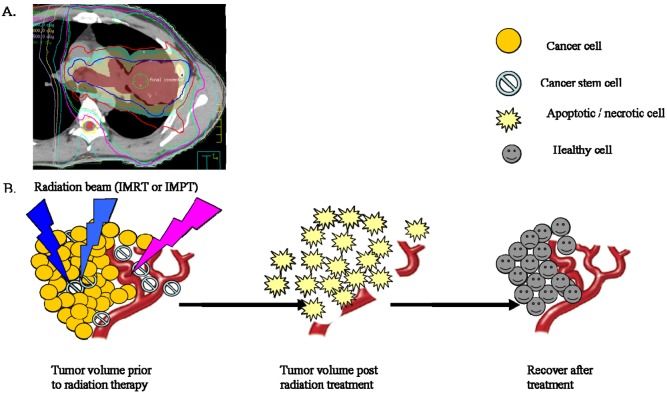
(**A**). Plan for intensity-modulate radiation therapy (IMRT) with dose escalation to gross tumor. (**B**). Concept of a new treatment plan that would be aimed at both the tumor bulk (non-stem cells) and the stem cell niche (CSCs). Biological imaging is applied to discriminate areas with CSCs from areas with non-stem cells. IMRT or intensity-modulated proton therapy allows delivery of customized dose distributions to the tumor bed; lower doses/shorter intervals between fractions (blue) for the tumor bed with higher doses/longer intervals between fractions (purple) for the CSC region could be combined with other treatment modalities such as surgery, chemotherapy, or gene therapy. This new concept would allow complete eradication of the tumor bed.

Conventional fractionated radiation therapy has evolved over more than a century as a means of delivering radiation dose over a period of time. Current standard treatment plans aim to deliver a homogenous dose over the tumor bed, assuming that CSCs are randomly distributed and therefore will be effectively removed by this method (Figure 2). Given our preliminary understanding of CSCs in their ability to recognize and repair radiation damage, to repopulate during the treatment period, and to recover from radiation damage between fractions, it is important that this concept be looked at wisely as it may change the way radiation is delivered. It might be possible that low radiation doses are given to non-CSCs while higher radiation doses are given to CSCs as this is believed to reflect their different intrinsic DNA repair efficiencies. Additionally, the time interval between radiation dose fractions might be different for CSCs *vs*. non-CSCs to account for the slow cycling of CSCs (Figure 3). It would be interesting to explore whether the following changes will be beneficial to cancer treatment: higher dose per fraction to overcome radiation resistance of CSCs and longer interval between fractionation to account for the slow cycling of CSCs and allow CSCs going through asymmetric division into less committed CSCs.

Radiation therapy, especially using photon energy has been a central player in the management of lung and esophageal cancer for a long time. Only recently, proton therapy has been used in lung and esophageal cancers due to its superior physical characteristics such that the proton beam can deliver a much higher therapeutic dose to certain depth without any exit dose; therefore, significantly reduce the radiation delivered to normal lung tissues and other nearby structures [122-123]. The selection of proton in cancer management becomes important because it addresses the pitfall in management of local failure in that an inadequate dose is given to the tumor bed because of toxicity concerns. Given its relatively new application in cancer management, there is little evidence regarding the applicability of proton therapy to CSCs. However, our preliminary studies have shown that proton therapy preferentially targets CSCs and increases ROS levels in treatment of resistant NSCLC to a greater extent than photons of the same dose [124]. This is a novel observation and would require further study. In addition, it would be interesting to see if similar observations are seen for other types of lung and esophageal tumors.

Given that CSCs share many of the same features as normal stem cells, there is a potential risk of killing normal stem cells while targeting CSCs. Therefore, it is important to further characterize the similarities and differences between these two types of stem cells and use new therapeutic approaches to selectively target CSC-specific pathways. One method is to target specific components of the signal transduction pathways that are highly active in CSCs, but not in normal stem cells, much like the strategy employed by therapeutics like trastuzumab (Herceptin^®^) in solid breast tumors [125]. An example of exploiting differences in CSC signaling is telomerase expression, which is repressed in most somatic cells but observed in stem cells as well as a high percentage of human cancers. It has recently been shown that telomerase expression is increased in radioresistant esophageal CSCs [100].

The observation that telomerase expression occurs in CSCs has important implications for developing therapeutic applications using telomerase. Since telomerase expression was believed to be restricted to CSCs, targeted strategies could be useful for therapy. Several groups have begun developing oncolytic Adenoviruses targeted to CSCs in the context of telomerase. Zhang and her colleagues engineered a telomerase-specific oncolytic Adeno vector carrying the apoptotic tumor necrosis factor-related apoptosis-inducing ligand under control of the hTERT promoter and cytomegalovirus early vector. This vector preferentially targeted radioresistant esophageal CSC-like cells and sensitized esophageal cancer to radiation [100]. However, the mechanism of tumoricidal effect of the oncolytic Adeno vector targeting the radioresistant cancer cells remains to be characterized.

Over its history, radiation oncologists have used local tumor control as an endpoint for radiotherapy with particular emphasis on the changes in tumor volume after therapy. This change in tumor volume is largely governed by the death of non-CSCs, while a subpopulation of CSCs might exist. Given what is known about the resilient growth properties of CSCs after radiation and that it only takes a very small number of CSCs, it is theoretically possible for a single surviving CSC to initiate a recurrent tumor. It is important to make a distinction that the response of the tumor cell bulk does not reflect the response of stem cells. Therefore, it might be wise to consider the absolute CSC control as the endpoint for radiotherapy. In that way, future development of new treatment planning and the assessment of local tumor control should be measured by the complete eradication of the subpopulations of CSCs.

Lastly, one cannot deny that combined-modality treatment approach has become standard and resulted in modest gain in the survival of lung and esophageal cancer patients. Therefore, the development of therapeutic agents specifically targeting CSCs coupled with a revised radiation treatment protocol will no doubt favorably alter the current poor prognosis of cancer patients. In this way, new treatments to combat lung and esophageal cancer will hopefully help to overcome the dismal outcome seen in these cancer patients.

## 5. Conclusions

This article gives an overview of the current literature regarding lung and esophageal CSCs and radiation resistance of CSCs. The consensus of opinion appears to be that there is little evidence of esophageal CSCs, in contrast to lung CSCs. While, at present, evidence continues to mount to support a CSC hypothesis, the extent of the remaining self-renewal capacity of lung and esophageal CSC populations after fractions of radiation is largely unknown. Further translational study to identify CSCs and analyze its behaviors will help researchers to explore CSCs targeting therapy. New novel CSC-based approaches may overcome treatment resistance and improve clinical outcome for cancer patients.

## Figures and Tables

**Table 1.** Cell markers and signaling pathways of human lung and esophageal cancer stem cells.

**Cancer Types**	**CSC Markers**	**Signaling Pathways**	**References**

Lung	CD133	Wnt	[23,32,65-68]
	CD44	Hedgehog	[41,46]
	SP		[45-47]
	Oct-4		[52]
	ALDH		[58-60]

Esophageal	CD44		[42]
	SP		[46-47]
	Oct-4		[47]
